# A comparative exploration of monoamine neurotransmitter transport disorders: mechanisms, clinical manifestations, and therapeutic approaches

**DOI:** 10.25122/jml-2024-0398

**Published:** 2025-03

**Authors:** Rand Redwan Al Sari, Husna Irfan Thalib, Syeda Sobiah Imad, Sariya Khan, Shyma Haidar, Bayan Mohammed Khair Al Zoabi, Sahar Hamed Fadda, Samratul Fuadah, Hassan Abu Alwan, Abdullah Alghobaishi

**Affiliations:** 1 Department of General Medicine and Surgery, Batterjee Medical College, Jeddah, Saudi Arabia; 2 Department of Medicine and Surgery, AlFaisal University, Riyadh, Saudi Arabia; 3 Department of General Medicine Practice and Surgery, King Khalid University, Abha, Saudi Arabia; 4 King Fahad Armed Forces Hospital, Jeddah, Saudi Arabia

**Keywords:** DTDS, PKDYS2, monoamine neurotransmitter transporters, dopamine transporter, DAT, Dopamine Transporter, VMAT2, Vesicular Monoamine Transporter 2, DTDS, Dopamine Transporter Deficiency Syndrome, PKDYS1, Infantile-Onset Parkinsonism Dystonia 1, PKDYS2, Infantile-Onset Parkinsonism Dystonia 2, DA, Dopamine, NE, Norepinephrine, CSF, Cerebrospinal Fluid, HVA, Homovanillic Acid, 5-HIAA, 5-Hydroxyindoleacetic Acid, SNP, Single Nucleotide Polymorphism, WES, Whole Exome Sequencing, EEG, Electroencephalography, ENMG, Electroneuromyography and Nerve Conduction Studies, MAO-A, Monoamine Oxidase A, MAO-B, Monoamine Oxidase B, AADC, Aromatic L-Amino Acid Decarboxylase, GAT-1, Gamma-Aminobutyric Acid (GABA) Transporter 1

## Abstract

Neurotransmitters play important roles in brain function, influencing cognition, movement, and behavior. Disruption in neurotransmitter biosynthesis, expression, transport, or function due to genetic mutations can lead to various neurological and psychiatric disorders with variable age of onset. Catecholamines like dopamine, norepinephrine, epinephrine, and serotonin are key monoamines transported by specific transporters, including the dopamine transporter (DAT) and the vesicular monoamine transporter 2 (VMAT2). Disorders that involve monoamine neurotransmitter transport include dopamine transporter deficiency syndrome (DTDS) and brain dopamine-serotonin vesicular disorders (PKDYS2). These rare syndromes manifest with movement disorders and neuropsychiatric symptoms. DTDS results from a mutation in the *SLC6A3* gene affecting dopamine reuptake, while PKDYS2 involves a mutation in the *SLC18A2* gene impairing the transport of dopamine and serotonin. This review provides a comparative analysis of the diagnostic approaches, the management strategies, and the outcomes for these distinct disorders.

## INTRODUCTION

Neurotransmitters are responsible for the relay of information in the brain. The cumulative actions and interaction of all the different types of neurotransmitters within and across neurons produce a range of functions, including cognition, movement, sensation, behavior, mood regulation, and the body's autonomic regulation. Neurotransmitters can be classified into canonical neurotransmitters, such as amino acids, monoamines, acetylcholine, and non-canonical neurotransmitters. Any impairment in the biosynthesis, expression, transport, and function of these neurotransmitters may arise due to various genetic mutations or exogenous factors. Such diseases usually manifest as various neurological and psychiatric symptoms and may have different age onset of presentation [1].

**Figure 1:**
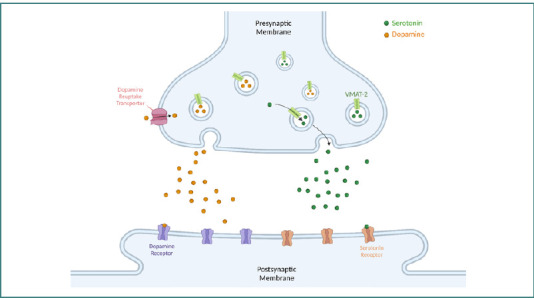
Overview of normal neurotransmission of dopamine and serotonin

Catecholamines, including dopamine (DA), norepinephrine (NE), epinephrine, and serotonin, are some of the most prevalent monoamine neurotransmitters, each containing a single amino group derived from different amino acids. Due to the structural similarity, many monoamine transporters can transport multiple types of monoamines, albeit with varying degrees of specificity. The expression of monoamine transporters is highly limited to dopaminergic neurons like those in the mesolimbic and nigrostriatal pathways [2]. Monoamine transporters are part of the solute carrier 6 (SLC6) family of transporters, and one example is the dopamine transporter (DAT) encoded by the *SLC6A3* gene. DAT transports dopamine from the synapse into the presynaptic neuron to regulate dopamine homeostasis [3]. Another transporter regulating dopamine movement is vesicular monoamine transporter 2 (VMAT2), encoded by the *SLC18A2* gene. It functions by translocating dopamine and serotonin into synaptic vesicles within the presynaptic neuron, as depicted in Figure 1 [4].

**Figure 2:**
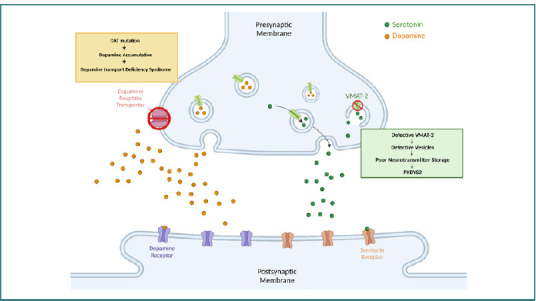
Monoamine neurotransmitter disorders

Monoamine neurotransmitter disorders are a heterogeneous group of disorders caused by defects in the dopaminergic system. They can be classified according to the etiology into primary, secondary, and unknown origin [5]. Dopamine dysregulation is characterized by symptoms including abnormal movement, neuropsychiatric features, and cognitive impairment. Dopamine transporter deficiency syndrome (DTDS) and brain dopamine-serotonin vesicular disorders are rare autosomal recessive primary monoamine neurotransmitter disorders, also known as infantile-onset parkinsonism-dystonia-1 (PKDYS1) and infantile-onset-parkinsonism-dystonia-2 (PKDYS2), respectively [3,4], as depicted in Figure 2. While both disorders involve dopamine dysregulation, they have distinct presentations and underlying mechanisms.

DTDS is caused by mutations in the *SLC6A3* gene, leading to a deficiency in the reuptake of dopamine. Patients may present with classic early-onset DTDS or atypical later-onset DTDS. In early-onset DTDS, the disease manifests in early infancy with nonspecific symptoms such as irritability and difficulty feeding, hypotonia, and delayed motor development. The spectrum of movement symptoms begins with hyperkinetic features, including chorea, dystonia, ballismus, and orolingual dyskinesia, evolving to features of parkinsonism-dystonia and finally to akinesia in late childhood/early adolescence. Despite the motor symptoms, patients present with good cognitive abilities and development. In contrast to classical DTDS, atypical DTDS is a more slowly progressive condition characterized by a later presentation in late adulthood with tremors, progressive bradykinesia, dystonic posturing, and variable tone [3].

Brain dopamine-serotonin vesicular transport disease is caused by mutations in the *SLC18A2* gene, resulting in impaired vesicular packing of catecholamines. Patients typically present in early childhood, and the presentation of this disease reflects the affected neurotransmitters with symptoms such as movement and muscle tone abnormalities such as dystonia, parkinsonism and variable muscle tone, sleep and mood disturbances, and autonomic instability such as diaphoresis, ptosis, and postural hypotension [4]. Due to an imbalance of the levels of certain neurotransmitters, various investigations, such as genetic and cerebrospinal fluid (CSF) analysis, can be conducted to diagnose these diseases, allowing for prompt management. This review will further discuss the most recent updates regarding the differences between these similar but distinct disorders, focusing on the diagnostic approach, management, and outcomes.

## MATERIAL AND METHODS

This literature review included many published studies until January 2024. The databases used include Google Scholar, PubMed, Springer, and BMC Library using the keywords ‘DTDS’, ‘PKDYS2’, ‘Monoamine Neurotransmitter Transporters’, and ‘Dopamine Transporter’. This review included studies in English that were central to the theme of this study. Articles in languages other than English were excluded. Data extraction was applied to differentiate patterns, trends, and key findings, contributing to a comprehensive understanding of the subject.

## APPROACH TO DIAGNOSIS

The diagnosis of DTDS and PKDYS2 involves a combination of clinical evaluation, laboratory investigations, and possible neuroimaging. It is confirmed by genetic testing.

### Clinical evaluation

The presentation of DTDS typically occurs in early infancy, within the first 6 months of life. The presentation is variable, as indicated in Table 1, but infants initially present with non-specific irritability and trouble feeding secondary to orolingual dyskinesia. Additionally, they may present with hyperkinesia, parkinsonism, or a mixed hypo- and hyperkinetic picture [6-10]. On the contrary, PKDYS2 manifests over a few weeks to months after birth. Individuals with PKDYS2 usually have developmental delays and exhibit a wide range of symptoms [11-13].

**Table 1:** Comparison between the classical presentations of DTDS and PKDYS2 [6-21]

Symptom	DTDS	PKDYS2
Hyperkinesia	✔	✘
Dystonia	✔	✔
Hypotonia	✔	✔
Parkinsonism	✔	✔
Oculogyric crisis	✔	✔
Global Developmental Delays	✔	✔
Sleep disorders	✘	✔
Mood disorders	✘	✔
Autonomic dysfunction	✘	✔

### Laboratory investigations

CSF analysis is usually performed in both cases as an initial investigation. Other investigations, such as urinary neurotransmitter analysis, may also be carried out. However, the analysis of monoamines or their metabolites in urine is not reliable for diagnosis, as the levels of metabolites of neurotransmitters in urine vary from those in CSF. The diagnostic test of choice is a genetic panel such as whole exome sequencing (WES) [4].

### CSF analysis via liquid chromatography

Normally, dopamine and serotonin are catabolized into homovanillic acid (HVA) and 5-hydroxy-indoleacetic acid (5-HIAA), respectively. Hence, these metabolites are used as indirect measures of neurotransmitter turnover in the brain [8]. In the case of DTDS, the function of the dopamine transporter (DAT) is affected such that the reuptake of dopamine by the presynaptic nerve endings is impaired. Dopamine that accumulates in the synaptic clefts is broken down to HVA, leading to the characteristically elevated HVA levels in CSF [7,8,11]. However, because DAT does not affect the serotonin pathway, the levels of 5-HIAA, the main metabolite of serotonin breakdown, are normal. Therefore, another useful finding is an HVA to 5-HIAA ratio > 4 [8,9,11]. However, in the case of PKDYS2, CSF is usually conducted with other diagnostic tests. Individuals with this condition have normal CSF neurotransmitters, with HVA and 5-HIAA levels within normal limits [12]. However, in rare cases, CSF neurotransmitters are mildly elevated with 5-hydroxyindoleacetic acid, but upon second analysis, it is common to have returned to the normal range [17]. Analysis of CSF remains challenging and may even be misleading if the sample collection and handling techniques are not in accordance with the strictly established protocols. It must be noted that the levels of HVA and 5-HIAA can be affected by several different groups of drugs [15]. Therefore, it is essential to obtain a thorough history from the parents or caretakers and review medical records to consider the effect of these drugs on the results of the CSF analysis.

### Genetic testing

In addition to the clinical evaluation and analysis of CSF, genetic testing must be conducted using genetic panels for dystonia disorders, single nucleotide polymorphism (SNP), and WES [15]. The diagnosis of DTDS is confirmed by either biallelic loss-of-function pathogenic variants in *SLC6A3* or heterozygous dominant-negative *SLC6A3* pathogenic variants known to cause autosomal dominant DTDS [10].

Meanwhile, the diagnosis of PKDYS2 is confirmed by detecting homozygous biallelic pathogenic variants in *SLC18A2* [13]. Some of the reported homozygous variants of *SLC18A2* confirmed by WES include c.1107dup, p.(Val370Serfs*91), c.1160C>T p.(Pro387Leu), c.710C>A p.(Pro237His, (c.876_883del p.Tyr293Hisfs*43), c.710C>T (p.Pro237His), *SLC18A2* (p.Pro237His) and *SLC18A2* c.1160C>T [11-13,17,18]. Additionally, a study reported the presence of two heterozygous variants in two different genes. These heterozygous variants by WES included *KMT2D*, c.13839+3C>A, and *CACNA1E*, c.3214G>A p. Val1072Il [13].

### Other laboratory investigations

Some other laboratory investigations can be obtained but are not mandatory to establish the diagnosis.

*Serum creatinine*: In the case of DTDS, serum creatinine kinase levels can be obtained. They are usually found to be elevated and are exacerbated by dystonic crises [8].

*Urinary neurotransmitter analysis*: In DTDS, assessing HVA in urine may be useful initially as it is a non-invasive test. HVA to creatinine ratio may also be measured and is usually elevated to greater than 15 μmol/mmol [7,8]. Similarly, in PKDYS2, monoamine metabolites HVA and HIAA in urine are consistently found in high concentrations [11,13].

*Serum prolactin*: In both disorders, serum prolactin levels may be obtained. Normally, dopamine functions as an inhibitor to prolactin release; therefore, elevated serum prolactin levels may raise suspicion of dopamine deficiency, especially if associated with galactorrhea [15].

### Neuroimaging

Although not essential for diagnosis, supportive imaging may also be obtained. A dopamine transporter (DAT) scan, performed after administering a DAT ligand named ioflupane (123I-2β-carbometoxy-3β-[4-iodophenyl]-N-[3-fluoropropyl] nortropane), reveals decreased or absent DAT activity in the basal ganglia in patients with DTDS [8,10]. In patients with PKDYS2, brain MRI with abnormally high T2 signal intensity with restricted diffusion in the dorsal brainstem can be noted [13]. Other subtle changes, such as cerebral atrophy, cortical volume loss, and mild ventricular enlargement, are also occasionally observed [12]. In one study, the MRIs of patients with DTDS showed delays in myelination and prominence of the external frontotemporal subarachnoid [8].

### EEG and EMG

Electroencephalography (EEG) and electromyography and nerve conduction studies (ENMG) have always been found to be normal in both DTDS and PKDYS2 [16,18,19].

### Differential diagnosis

It is important to correctly assess patients with DTDS because a misdiagnosis of cerebral palsy or other disorders is often incorrectly reached [7,8]. Similarly, PKDYS2 is often confused with Menkes disease due to the presence of the *ATP7A* gene. *ATP7A* gene mutation occurs in Menkes disease, which leads to dysfunction in copper homeostasis. However, if the copper levels and ceruloplasmin concentrations are normal, this gene mutation could be associated with PKDYS2 [16]. Sometimes, patients with oculogyric crises and dystonia are misdiagnosed with epilepsy [13]. Additionally, neurotransmitter disorders such as aromatic l-amino acid decarboxylase deficiency (OMIM 608643) could be identified as possible differential diagnoses [12]. Furthermore, hypoxic-ischemic encephalopathy, cerebral palsy, other movement disorders, and paroxysmal conditions could also mimic this disorder [11,20,21]. Moreover, DTDS and PKDYS2 are both important differentials with similar presentations.

## MANAGEMENT

The management of PKDYS2 involves various treatment modalities. Multiple dopaminergic drugs (levodopa, pramipexole, and methylphenidate) are commonly used to control static tremors [14,16]. However, when clinical responses to these agents are insufficient, medications that enhance neurotransmission—such as serotonergic drugs and tetrahydrobiopterin (BH4) supplementation—are recommended [15]. In contrast, patients with DTDS are not treated with anticholinergics and benzodiazepines. One study reported that dopamine receptor agonists, specifically ropinirole and pramipexole (also used in adult-onset Parkinson's disease), showed some benefit in patients with DTDS. However, there are limitations, and more information is needed to establish the pediatric dose, side effects, and long-term outcomes [20]. An overview of the pharmacotherapies used for PKDYS2 and/or DTDS, along with the specific clinical symptoms they address, is presented in Table 2 [11].

**Table 2:** Pharmacological agents and their corresponding clinical presentations in dopamine-related disorders

Drug	Clinical feature	Disorder
L-Dopa	Dopa-responsive dystonia, classically ‘evening equinus’, diurnal fluctuation Truncal hypotonia, dystonia, developmental delay, seizures Hypotonia, hypokinesia, rigidity, chorea, dystonia, oculogyric crisis Axial hypotonia, dystonia, oculogyric crisis, diurnal fluctuation, CP-like presentation Bulbar dysfunction, dyskinesia, tremor, dystonia, choreoathetosis Parkinsonism–dystonia, hypokinesia or bradykinesia, rigidity, diurnal variation Focal or generalized dystonia with crises; severe parkinsonism, hypotonia, oculogyric crises, tremor, ptosis, hypersalivation, autonomic disturbance Mild learning difficulties, hyperactivity, sleep disturbance, distinctive facial features, hypoplastic middle 5th phalanges Axial hypotonia, oculogyric crises, parkinsonism, tremor, facial dyskinesia, ptosis, bulbar dysfunction, sleep disturbance	AD GCH deficiency (Segawa’s syndrome) [22,23] AR GCH deficiency [24,25] PTPS deficiency [24,25] SRD deficiency [26-28] DHPR deficiency [24,29] TH deficiency type A [30] TH deficiency type B [30] PITX3 deletion [31] Brain dopamine –serotonin vesicular transport disease [32]
Supplementation enzyme cofactors: BH4 supplementation	Truncal hypotonia, dystonia, developmental delay, seizures Hypotonia, hypokinesia, rigidity, chorea, dystonia, oculogyric crisis Axial hypotonia, dystonia, oculogyric crisis, diurnal fluctuation, CP-like presentation Bulbar dysfunction, dyskinesia, tremor, dystonia, choreoathetosis	AR GCH deficiency [24,25] PTPS deficiency [24,25] SRD deficiency [26-28] DHPR deficiency [24,29]
5-HTP	Truncal hypotonia, dystonia, developmental delay, seizures Hypotonia, hypokinesia, rigidity, chorea, dystonia, oculogyric crisis Axial hypotonia, dystonia, oculogyric crisis, diurnal fluctuation, CP-like presentation Bulbar dysfunction, dyskinesia, tremor, dystonia, choreoathetosis	AR GCH deficiency [24,25] PTPS deficiency [24,25] SRD deficiency [26-28] DHPR deficiency [24,29]
Dopamine agonists	Hypotonia, hypokinesia, rigidity, chorea, dystonia, oculogyric crisis Bulbar dysfunction, dyskinesia, tremor, dystonia, choreathetosis. Hypotonia, oculogyric crisis, hypokinesia, chorea, dystonia, bulbar dysfunction Axial hypotonia, oculogyric crises, parkinsonism, tremor, facial dyskinesia, ptosis, bulbar dysfunction, sleep disturbance. Feeding difficulties, irritability, axial hypotonia, and dyskinetic movement disorder, a progressive dystonic and dyskinetic movement disorder with eye involvement	PTPS deficiency [25] DHPR deficiency [33] AADC deficiency [34,35] Brain dopamine –serotonin vesicular transport disease [8] Dopamine transporter deficiency syndrome [8]
Anticholinergic medications	Generalized and segmental dystonia	Dopamine transporter deficiency syndrome
GABAergic agents	Dystonia	Dopamine transporter deficiency syndrome

L-Dopa, Levodopa; AD, Autosomal Dominant; AR, Autosomal Recessive; GCH, GTP Cyclohydrolase; PTPS, 6-Pyruvoyl-Tetrahydropterin Synthase; SRD, Sepiapterin Reductase Deficiency; DHPR, Dihydropteridine Reductase; TH, Tyrosine Hydroxylase; PITX3, Paired-like Homeodomain Transcription Factor 3l; BH4, Tetrahydrobiopterin; 5-HTP, 5-Hydroxytryptophan; AADC, Aromatic L-Amino Acid Decarboxylase; CP, Cerebral Palsy.

Other treatments, such as monoamine oxidase type A (MAO-A) and MAO type B (MAO-B), are also used as an alternative/adjunct therapy, especially in conditions unresponsive to L-dopa or to enable the reduction of L-dopa dose [11]. Selective serotonin-reuptake inhibitors (SSRIs) are also used in childhood neurotransmitter disorders. For example, fluoxetine has been used in aromatic L-amino acid decarboxylase (AADC) deficiency [36] because it binds with high-affinity to the serotonin transporter, inhibiting reuptake inhibition of synaptic serotonin and enhancing neurotransmission with minimal effects on norepinephrine and dopamine uptake, and displaying minimal binding to postsynaptic receptors [37,38]. The use of MAO-B inhibitors (selegiline) in conjunction with SSRIs can lead to a serotonergic crisis with clinical features of dyskinesia, confusion, and hypertension and is managed with the combined withdrawal of both medications. In addition, cerebral folate deficiency has been observed in DHPR and AADC deficiencies, and folinic acid supplementation has been successful in restoring cerebral folate levels—thereby helping to prevent further neurological impairment [11]. Furthermore, literature has shown that PKDSY2 often necessitates additional supportive care, including nutritional and feeding support for oral feeding difficulties, alternative communication devices when needed, and regular physical therapy to reduce the risk of contractures and fractures. Focal botulinum toxin injection could be used for contractures, and prophylactic antibiotics, along with chest physiotherapy, can prevent further pulmonary infections. Melatonin or other sedatives may be employed for sleep disturbances. Moreover, patients should benefit every 6 to 12 months from surveillance and evaluation of nutrition, swallowing, speech-language, and physiotherapy evaluation for postural and tone issues [3]. In contrast, DTDS usually presents with episodes of dystonic crisis requiring emergency management to prevent complications such as extreme pain, severely disrupted sleep pattern, abnormal posture preventing seating, rhabdomyolysis, myoglobinuria, dystonia-triggered diaphragmatic splinting, and respiratory failure. Hospitalization is often required for aggressive muscle relaxation and sedation to resolve status dystonicus, using treatments such as continuous intravenous midazolam infusion, morphine, and, in some instances, general anesthesia [39].

## OUTCOME

Management of DTDS remains palliative, focusing on symptom control rather than cure. As mentioned previously, early-stage treatments are mainly symptomatic and were shown to have limited efficacy. Early-stage treatments for chorea and dyskinesia include tetrabenazine and benzodiazepines, while dopamine agonists such as pramipexole and ropinirole are used for dystonia, similar to PKDYS2. Unfortunately, these interventions often have limited efficacy, and there is generally poor response to levodopa. Gabapentin has shown some benefit in reducing stiffness and hyperkinesia [19]. For focal dystonia, botulinum toxin injections and orthopedic surgeries for severe contractures have been employed. In some older patients, neurosurgical approaches like deep brain stimulation and intrathecal baclofen have been attempted, albeit with limited success [3]. Managing the symptoms of PKDYS2 encompasses a wide variety of medications, and each of these interventions has its benefits and potential harms. Pramipexole is one of the widely used therapeutic interventions to treat dystonic posturing and motor symptoms related to PKDYS2, as mentioned before. In a 15-month-old boy who was diagnosed with PKDYS2, initial treatment was started with pramipexole (0.005 mg/kg/day). This improved dystonic posture, but when the dose was increased to 0.01 mg/kg/day, it worsened hypotonia, irritability, and sleep difficulties. Similarly, an 8-month-old boy was diagnosed with PKDYS2 (SLC18A2 c.710C>A; p. Pro237His) and additional genetic variants. L-DOPA-carbidopa caused dyskinesia, and pramipexole induced hyperkinetic movements at higher doses, leading to minimal long-term improvement and severe motor delays. Therefore, it is to be noted that although initial lower doses of pramipexole were found to be effective, higher doses most often led to side effects [13]. In another report involving male twins, one twin died at age 10 while the surviving twin, treated with pramipexole, exhibited only mild improvements in alertness, communication, and eye movements [18]. L-DOPA/carbidopa can provide modest improvements in some cases; however, its use may lead to adverse effects such as facial dyskinesia and choreiform trunk movements. Other therapeutic strategies are also used to manage specific symptoms and reduce side effects. These include trihexyphenidyl for dystonia, baclofen to reduce spasticity and muscle rigidity, 5-hydroxytryptophan to support neurotransmitter balance, and Sinemet—which combines carbidopa and levodopa for increased efficacy of treatment. For example, in a 10-year-old girl with PKDYS2 diagnosed at 4 months, L-DOPA/carbidopa initially provided slight improvement. However, switching to pramipexole (0.18 mg twice daily) combined with baclofen significantly improved motor functions, although behavioral issues required dose adjustments [13]. Together, these treatment strategies offer a multi-disciplinary approach to managing the disease. Nonetheless, careful monitoring and dosage adjustments are vital to control therapeutic benefits as well as adverse effects.

The quality of life in these patients is significantly impacted by both the treatment interventions and the severity of their symptoms. Day-to-day functioning is impaired as a result of motor and speech delays, recurrent dystonia, and medical complications, including frequent infections and aspiration pneumonia. Bulbar dysfunction and feeding difficulties necessitate nutritional support for both DTDS and PKDYS2, often via gastrostomy feeding, due to poor weight gain and risk of aspiration pneumonia. Respiratory complications such as frequent chest infections pose significant mortality risks. Notably, the literature reveals that children diagnosed with DTDS have succumbed to cardiorespiratory complications, with a mean age of death of 10.4 years. The long-term prognosis for early-onset DTDS remains guarded, with high morbidity and premature mortality, although some individuals have survived into their fourth decade of life [19]. Some patients also need G-tube feeding and tracheostomy due to the severe complications of the disease. The long-term prognosis of patients is directed by the progressive nature of the disease, generally involving worsening motor symptoms and developmental delays. Treatment effectiveness varies greatly. In patients with PKDYS2, the severity of symptoms, especially in patients with remarkable genetic mutations like the p.Pro237His variant, correlates with poorer outcomes and higher mortality rates than other variants such as p.Pro387Leu [13]. Moreover, side effects from medications and recurrent infections can lead to complications in management and worsen prognosis. This underscores the importance of effective management of side effects. Genetic factors, including the presence of variants like *CACNA1E* and *KMT2D*, further complicate the disease course and prognosis, necessitating tailored treatment approaches for each patient [40,41].

Future directions in the treatment of DTDS and PKDYS2 are dependent on ongoing research and technological advancements, which promise significantly improved patient outcomes. The promising preclinical and clinical outcomes of recombinant adeno-associated virus (rAAV2-AADC) gene therapy for Parkinson's disease (PD) and AADC deficiency have motivated researchers to develop gene therapies for DTDS. Likewise, research focused on targeted treatments addressing specific *SLC18A2* mutations and individualized strategies for PKDYS2 can help in developing more effective therapies with fewer side effects [42]. Genetic insights regarding additional variants and their impact on DTDS and PKDYS2 are essential for guiding precise treatment approaches, which might potentially lead to better outcomes. Personalized medicine strategies are becoming increasingly crucial in drug development, especially for inherited neurodegenerative disorders where current treatments only focus on managing symptoms and providing palliative care. Through the innovative use of induced pluripotent stem cell (iPSC)-derived neuronal models and mouse models, a deeper insight into the underlying mechanisms of DTDS can be gained, which will aid in the exploration of potential therapies for this condition, which is particularly resistant to standard treatments [6].

A recent study using iPSC-derived neurons and knockout mouse models accurately replicated key features of DTDS, such as impaired DAT activity, elevated dopamine metabolites, and neurodegeneration. The mouse model mirrors important motor symptoms seen in human patients, progressing from early hyperkinesia to late-stage parkinsonism [21].

The iPSC-based platform offers several advantages, allowing researchers to study patient-specific DAT mutations in a relevant human neuronal system. This includes variants that are lethal when studied in other models, such as L368Q. By combining insights from iPSC and murine models, a further understanding of how DAT dysfunction leads to neurodegeneration can be obtained. Notably, both models showed reduced levels of MAO-A and MAO-B enzymes in dopamine catabolism pathways, suggesting compensatory changes in response to impaired dopamine reuptake [21].

Technological advancements, such as improved diagnostic tools through advanced genetic testing and personalized medicine strategies, are aimed at enhancing diagnosis and treatment planning, which allows for more tailored and effective interventions for both DTDS and PKDYS2. Furthermore, advanced imaging and biomarker studies can help monitor disease progression and treatment efficacy with enhanced precision. This can help in providing valuable insights into the disease course. Novel treatments are emerging, with interventions exploring neurotransmitter-modulating agents that minimize side effects, such as serotonergic drugs and tetrahydrobiopterin (BH4) supplementation for PKDYS2 [43].

Current literature supports the need for a clinically viable gene therapy approach using AAV2 vectors targeted to the DAT-expressing brain regions, demonstrating therapeutic efficacy without neurotoxic side effects in preclinical models. Future research should explore synaptic neurotransmission and cognitive behaviors using advanced techniques like brain organoids and optogenetics [21]. Gene therapy, especially AAV-mediated gene transfer, has shown good potential in early clinical trials, offering critical improvements in motor symptoms and overall prognosis by identifying and addressing the underlying genetic causes. Four children diagnosed with AADC deficiency recently underwent AAV-mediated transfer of the human AADC gene through bilateral stereotactic injection to the putamen. This has shown promising results, with improved motor symptoms in all the patients, clinical safety, and increased dopamine and serotonin levels following gene transfer [44].

Larger clinical trials will be more informative as to the relative benefit of gene therapy in this group of patients. Challenges such as accurate neurosurgical targeting and optimal vector dosing need careful consideration to facilitate clinical translation. Selection of suitable patients based on genotype, age, and disease stage will be critical for maximizing therapeutic efficacy.

Future directions for DTDS and PKDYS2 focus on refining genetic diagnostic techniques and developing more effective, targeted therapies. Genetic counseling is vital for affected families, with options for prenatal and preimplantation testing to identify at-risk individuals. Other strategies include research into catechol-O-methyltransferase (COMT) inhibitors as well as other innovative treatments, which could provide new therapeutic strategies for effectively managing disease symptoms [25,45].

## ADVANCES IN PHARMACOLOGICAL CHAPERONING: FUTURE THERAPEUTIC DIRECTIONS

Recent research has highlighted pharmacological chaperoning as a promising approach to restoring the function of neurotransmitter transporters affected by genetic mutations. For example, Asjad *et al*. in 2017 showed that pharmacological chaperones can effectively stabilize and restore the activity of DAT variants, which are linked to DTDS. This approach works by helping defective transporter proteins fold properly and reach their correct location, ultimately boosting their function [46]. Similar success has also been seen in drosophila models with other members of the SLC6 transporter family, like GABA Transporter 1 (GAT-1), where pharmaco-chaperoning has helped to rectify childhood epilepsy variants related to treatment-resistant epilepsy [47,48]. These findings demonstrate the therapeutic potential of chaperones and open up new possibilities for treating transporter-related dysfunctions in various neurological and neurodevelopmental disorders.

## CONCLUSION

Monoamine transport disorders, including DTDS and PKDYS2, pose significant clinical challenges due to their rarity and symptomatic complexity. Early and accurate diagnosis facilitated by laboratory investigations, genetic testing, and comprehensive clinical evaluation is crucial to initiate appropriate management strategies. Although current therapeutic options primarily focus on improving symptoms and quality of life, further research and technological advancements are required to enhance long-term prognosis and explore potential therapies. According to the literature, future goals for managing patients with DTDS and PKDYS2 include refining genetic diagnostic techniques and developing more effective multidisciplinary targeted therapies to improve outcomes. Moving forward, the literature supports the need for clinically viable gene therapy and explores personalized medicine strategies for optimizing treatment outcomes in individuals affected by these challenging neurological disorders.

## Personal thanks

The authors thank Dr. Abdullah Alghobaishi for critically guiding and reviewing this article until publishing.
